# Low distribution of genes encoding virulence factors in *Shigella flexneri* serotypes 1b clinical isolates from eastern Chinese populations

**DOI:** 10.1186/s13099-017-0222-9

**Published:** 2017-12-16

**Authors:** Wenting Fan, Huimin Qian, Wenkang Shang, Chen Ying, Xuedi Zhang, Song Cheng, Bing Gu, Ping Ma

**Affiliations:** 1 Medical Technology School, Xuzhou Medical University, Xuzhou, 221004 China; 2 Department of Acute Infectious Disease Prevention and Control, Jiangsu Provincial Center for Disease Prevention and Control, Nanjing, 210029 China; 3 Department of Physiology, Xuzhou Medical University, Xuzhou, 221004 China; 4 Department of Laboratory Medicine, Xuzhou Infectious Disease Hospital, Xuzhou, 221004 China; 5 Department of Laboratory Medicine, Affiliated Hospital of Xuzhou Medical University, Xuzhou, 221002 China; 6 Department of Laboratory Medicine, Affiliated Hospital of Xuzhou Medical University, No. 99 West Huaihai Road, Xuzhou, 221006 China

**Keywords:** *Shigella*, Virluence gene, Serotype, Clinical value

## Abstract

**Background:**

The ability of *Shigella* to invade, colonize, and eventually kill host cells is influenced by many virulence factors. However, there is no analysis of related genes in Jiangsu Province of China so far. *Shigella flexneri* was collected from 13 cities of Jiangsu Province through the provincial Centers for Disease Control (CDC) for analysis of distribution of major virulence genes (*ipa*H, *ipa*BCD, *ial*, *vir*F, *vir*B, *sig*A, *set*1A, *sep*A, *sat*, *pic*, *set*1B and *sen*) detected by PCR technology.

**Results:**

A total of 545 isolates received were confirmed as *S. flexneri* which belongs to 11 serotypes of *S. flexneri*, among which serotype 2a was the most predominant (n = 223, 40.9%). All isolates were positive for *ipa*H gene, followed by *sat* (94.1%), *sig*A (78.9%), *set*1B (78.0%), *pic* (77.6%), *set*1A (74.5%), *vir*F (64.8%), *sep*A (63.5%), *sen* (56.9%), *ipa*BCD (50.5%), *ial* (47.0%) and *vir*B (47.0%). The presence of virulence genes in different serotypes was distinct. The existence of virulence genes of serotype 1b was generally lower than other serotype-the positive rate for virulence genes was between 0.0 and 14.1% except for *ipa*H and *sat*. In addition, virulence genes also fluctuated in different regions and at different times in Jiangsu province. The result of analysis on the relationship between virulence genes of *S. flexneri* showed that the existence of virulence genes of *Shigella* could be well represented by multiplex PCR combination *ipa*H + *ial* + *set*1A, which had a high clinical value.

**Conclusions:**

The present study was designed to explore the prevalence of 12 *S. flexneri*-associated virulence genes. The data showed high diversity of virulence genes with regard to periods, regions and serotypes in Jiangsu Province of China.

**Electronic supplementary material:**

The online version of this article (10.1186/s13099-017-0222-9) contains supplementary material, which is available to authorized users.

## Background

Shigellosis is major health burden in many parts of the world. It is an acute invasive enteric infection caused by four members of *Shigella* species (*S. dysenteriae*, *S. flexneri*, *S. boydii*, and *S. sonnei*). Different serotypes for these species exist including more than 30 serotypes of *S. flexneri* which are categorized based on their O antigens, [1]. Although the role of shigellosis in contributing to childhood mortality has been decreased significantly over the past few years, there are still about 28,000 children younger than 5 years of age who died of shigellosis every year [2]. In a systematic review, [3] it was reported that due to low economic conditions and large population density in Asian countries, over 125 million *Shigella*-related infections led to 14,000 deaths per year. There are some factors contributing to the high prevalence of human *Shigella* infection. One reason for the high infection rate in some developing countries is the low sanitary conditions, knowing that *Shigella* spp. is transmitted via the fecal–oral route. Another important factor is that *S. flexneri* possess protective mechanisms that help it to survive even at high levels of acid in the stomach, which makes it highly infectious with only 10–100 microorganisms required to cause a disease [4].

In children, main symptoms of shigellosis vary from mild to severe which include: diarrhoea characterized by presence of blood in stool, abdominal cramping, fever, among other gastrointestinal complications. Its clinical phenotypes are determined by different virulence genes and the activity of immune system of the host. Among the many *Shigella* spp.—associated virulence factors, invasion plasmid antigen (ipa) B, C, D, and H as well as invasion-associated locus (ial) facilitates its penetration into intestinal cells [4]. As with gram-negative bacteria, these genes are important for S. *flexneri* because they are components of the type III secretion system (T3SS) which is important for *S. flexneri* and other gram-negative pathogenic or symbiotic bacteria in manipulating the host cell processes and establish a successful infection [5]. *Shigella* enterotoxin 1 (ShET-1), *Shigella* enterotoxin 2 (ShET-2) and shiga toxin (stx) are among virulence genes encoding *Shigella* enterotoxins. A group of genes mostly found in *S. flexneri* serotype 2 clinical samples encode ShET1, a 55 kDa protein complex [6, 7]. ShET2 has been reported in different species of *Shigella* [7]. The stx is produced exclusively by *S. dysenteriae* 1, but this species is rare in China [8]. The transcription of invasion-related genes is controlled by two proteins, virF and virB (InvE) which are derived from plasmids [9]. Finally, *Shigella* spp. harbors toxic factors like serine protease autotransporters of enterobacteriaceae (SPATE) of which there are two phylogenetic classes [10]. *Shigella* IgA-like protease homologue (sigA) and secreted autotransporter toxin (sat) belong to class 1 which are toxic to epithelial cells, while non-toxic SPATE class 2 toxins includes sepA, which facilitates intestinal inflammation and pic, a mucinase associated with colonization.

Although some studies reported the prevalence and distribution of *S. flexneri* virulence genes in some regions in China, investigations to dozen virulence genes of *Shigella* spp. mentioned above are still rare throughout the world, and to the best of our knowledge there is no report in China. To develop effective control strategies, it is important to conduct an epidemic study about *Shigella* in terms of its drug resistance and genetic features [11]. For this reason, we sought to explore the distribution profile and prevalence of 12 *Shigella*-related virulence genes obtained from patients with diarrhea in Jiangsu Province of China, and discussed the genetic diversity and clinical applications of these genes.

## Methods

### Collection of bacteria isolates

**Fig. 1:**
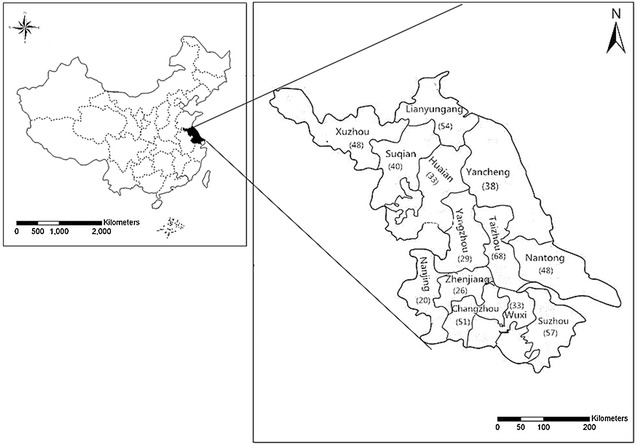
Regional distribution of *Shigella flexneri* in Jiangsu province

CDC-based real-time surveillance program in 13 cities of Jiangsu Province from 2010 to 2015 (Fig. 1) were conducted by collecting suspecting *Shigella* spp. isolates from different patients with either diarrhea or dysentery in different hospitals in 13 cities by using routine biochemical techniques. *Shigella* is a class B infectious disease in China. The bacteria detected in any local hospital must be reported to the provincial CDC by the city’s CDC. The study was conducted in collaboration with the Provincial CDC, so the collection of *Shigella* was the most comprehensive.

### Bacteria identification and serotyping

By use of Rapid ID32E strips (bioMérieux Corp., Singapore) and automated biochemical analyzer (Hitachi 917; Boehringer Mannheim, Japan), the collected samples were processed and screened. O and H antigens were examined using hyperimmune sera through slide agglutination test (Ningbo Tianrong Bio-pharmaceutical Company Limited), and thereafter, the serotypes were grouped according to the Kauffmann–White scheme.

### Polymerase chain reaction (PCR) assay for virulence genes

Qiagen DNA mini kit was used for the extraction of DNA in line with the manufacturer’s instructions. The polymerase chain reaction (PCR) assays were performed targeting virulence genes using previously reported primers listed in Table 1. A reaction mixture, Green Taq Mix (Vazyme, Nanjing, China) was prepared as per manufacturer’s guidelines. Amplification was done in a thermocycler programmed with the following sequence: a 5-min initial denaturation at 95 °C, then 30 cycles including a 50 s denaturation at 95 °C, annealing for 45 s (annealing temperature is shown in Table 1), and 72 °Cfor 1 min and a single final extension at 72 °C for 7 min. For each virulence gene detected, a representative amplicon was sequenced to confirm that the gene was amplified by its specific primer.

**Table 1:** Primers used in this study

Target gene	Primer	Sequence (5′–3′)	Annealing	Size (bp)
*ipa*H	*ipa*H-F	TGGAAAAACTCAGTGCCTCT	55 °C [12]	423
	*ipa*H-R	CCAGTCCGTAAATTCATTCT		
*ial*	*ial*-F	GCTATAGCAGTGACATGG	55 °C [13]	320
	*ial*-R	ACGAGTTCGAAGCACTC		
*vir*B	*vir*B-F	CGATAGATGGCGAGAAATTATATCCCG	56 °C [14]	766
	*vir*B-R	CGATCAAGAATCCCTAACAGAAGAATCAC		
*vir*F	*vir*F-F	AGCTCAGGCAATGAAACTTTGAC	60 °C [15]	618
	*vir*F-R	TGGGCTTGATATTCCGATAAGTC		
*sig*A	*sig*A-F	CCGACTTCTCACTTTCTCCCG	58 °C [16]	430
	*sig*A-R	CCATCCAGCTGCATAGTGTTTG		
*sep*A	*sep*A-F	GCAGTGGAAATATGATGCGGC	58 °C [17]	794
	*sep*A-R	TTGTTCAGATCGGAGAAGAACG		
*pic*	pic-F	ACTGGATCTTAAGGCTCAGGAT	58 °C [17]	572
	pic-R	GACTTAATGTCACTGTTCAGCG		
*sat*	sat-F	TCAGAAGCTCAGCGAATCATTG	59 °C [16]	930
	sat-R	CCATTATCACCAGTAAAACGCACC		
*set*1A	*set*1A-F	TCACGCTACCATCAAAGA	57 °C [13]	309
	*set*1A-R	TATCCCCCTTTGGTGGTA		
*set*1B	*set*1B-F	GTGAACCTGCTGCCGATATC	57 °C [13]	147
	*set*1B-R	ATTAGTGGATAAAAATGACG		
*ipa*BCD	*ipa*BCD-F	GCTATAGCAGTGACATGG	59 °C [18]	500
	*ipa*BCD-R	ACGAGTTCGAAGCACTC		
*sen*	*sen*-F	ATGTGCCTGCTATTATTTAT	55 °C [13]	799
	*sen*-R	CATAATAATAAGCGGTCAGC		

### Statistical analysis

Statistical analyses were performed using SPSS 16.0 database software. Distribution of different virulence genes in serotypes, periods and regions were analyzed by the Chi square test, and statistical differences between groups were considered to be significant for p < 0.05.

## Results

### *S. flexneri* isolated during the study period

Altogether 545 isolates were confirmed as *S. flexneri* during the 6-year period of investigation. These 545 strains were distributed in 13 cities of Jiangsu province. The prevalence ranked as follows: Taizhou (n = 68, 12.5%), Suzhou (n = 57, 10.5%), and Lianyungang (n = 54, 9.9%). What’s more, only 20 (3.7%) strains were from Nanjing, the capital of Jiangsu Province (Fig. 1). In the year variation of *S. flexneri*, it was found that the most of the *S. flexneri* were isolated in 2012, and there was a trend of decrease in the following years (Table 2).

**Table 2:** Year wise distribution of serotypes of *S. flexneri*

	2015	2014	2013	2012	2011	2010	Total
F2a^A^	35	32	41	47	24	44	223
F1a	9	13	28	25	4	6	85
F1b	19	12	10	20	2	1	64
F2b	14	13	19	21	12	27	106
Fx	3	11	4	0	0	12	30
F4c	1	0	8	2	2	5	18
Fy	0	3	2	1	1	0	7
F4	0	0	2	0	0	0	2
F6	0	0	0	0	0	5	5
F3b	0	1	0	1	0	0	2
F4a	0	0	0	3	0	0	3
Total	81	85	114	120	45	100	545

^A^F2a: *S. flexneri* serotype 2a, the same below

### Serotypes of *S. flexneri*

All the 545 isolates of *S. flexneri* belonged to 11 serotypes of *S. flexneri*. Of these, *S. flexneri* serotype 2a was the most predominant (n = 223, 40.9%) compared with the other serotypes. 2a, 2b, 1a, 1b, x and 4c were the six most frequently isolated serotypes, accounting for 96.5% of all *S. flexneri*, while other serotypes accounted for less than 1.5%. Six major serotypes had an obvious fluctuation over time (Table 2). The other infrequently observed serotypes, including Y, 4, 4a, 3b and 6, were only found in a small amount within a certain period of one or 2 years.

### Prevalence of virulence genes

#### Invasion-associated genes

The detection of invasion-associated genes in 545 *S. flexneri* showed that *ipa*H had the highest frequency (100%) followed by followed by *ipa*BCD (50.5%) and *ial* (47.0%) (Table 3). There were 253 (46.4%) strains with three invasive genes. The invasion-associated genes had serotype differences. The *ial* gene was detected in 66.7% of 18 *S. flexneri* 4c isolates, 60.1% of 223 *S. flexneri* 2a isolates, 50.9% of 106 *S. flexneri* 2b isolates, 32.9% of 85 *S. flexneri* 1a isolates and 56.7% of 30 *S. flexneri* x isolates. However, the *ial* gene was just found in 1.6% of 64 *S. flexneri* 1b. The *ipa*BCD gene was detected in 66.7% of *S. flexneri* 4c, 62.8% of *S. flexneri* 2a, 55.7% of *S. flexneri* 2b, 36.5% of *S. flexneri* 1a, 66.7% of *S. flexneri* x and none of the *S. flexneri* 1b.

**Table 3:** Presence of virulence genes in different serotypes of *S. flexneri*

	F2a (%)	F2b (%)	F1a (%)	F1b (%)	FX (%)	FY (%)	F4c (%)	F4 (%)	F4a (%)	F3b (%)	F6 (%)	Total (%)
*ipa*H	100.0	100.0	100.0	100.0	100.0	100.0	100.0	100.0	100.0	100.0	100.0	100.0
*ial*	60.1	50.9	32.9	1.6	56.7	71.4	66.7	0.0	33.3	0.0	80.0	47.0
*ipa*BCD	62.8	55.7	36.5	0.0	66.7	71.4	66.7	100.0	33.3	0.0	100.0	50.5
*ipa*H + *ial* + *ipa*BCD	60.1	50.0	32.9	0.0	56.7	71.4	61.1	0.0	33.3	0.0	80.0	46.4
*vir*F	75.8	73.6	52.9	1.6	93.3	100.0	77.8	100.0	100.0	50.0	100.0	64.8
*vir*B	60.1	46.2	34.1	0.0	66.7	57.1	66.7	100.0	33.3	0.0	100.0	47.0
*vir*F + *vir*B	58.7	40.6	32.9	0.0	66.7	57.1	61.1	100.0	33.3	0.0	100.0	45.0
*sat*	93.3	93.4	97.6	98.4	100.0	100.0	88.9	100.0	100.0	100.0	0.0	94.1
*sig*A	91.9	84.0	87.1	1.6	100.0	85.7	88.9	100.0	66.7	0.0	100.0	78.9
*pic*	92.4	81.1	84.7	0.0	100.0	100.0	88.9	100.0	100.0	0.0	20.0	77.6
*sep*A	74.4	70.8	51.8	1.6	86.7	100.0	94.4	100.0	100.0	50.0	80.0	63.5
*set*1A	86.1	73.6	87.1	15.6	83.3	100.0	88.9	50.0	66.7	0.0	20.0	74.5
*set*1B	92.4	81.1	84.7	9.4	93.3	71.4	94.4	100.0	100.0	0.0	0.0	78.0
*set*1A + *set*1B	84.3	67.9	78.8	1.6	76.7	71.4	88.9	50.0	66.7	0.0	0.0	68.8
*sen*	68.6	57.5	44.7	14.1	73.3	71.4	77.8	100.0	66.7	50.0	60.0	56.9

### Regulatory genes

A total of 353 (64.8%) isolates tested positive for *vir*F genes while 256 (47.0%) isolates were positive for *vir*B genes (Table 3). 254 (45.0%) bacteria simultaneously had two genes. In addition, *vir*B gene existed in 66.7% of *S. flexneri* 4c and 66.7% of *S. flexneri* x, followed by *S. flexneri* 2a (60.1%), *S. flexneri* 2b (46.2%), *S. flexneri* 1a (34.1%) and *S. flexneri* 1b (0.0%). *vir*F gene was found in 93.3% of *S. flexneri* x, followed by *S. flexneri* 4c (77.8%), *S. flexneri* 2a (75.8%), *S. flexneri* 2b (73.6%), *S. flexneri* 1a (52.9%), *S. flexneri* 1b (1.6%).

#### SPATEs

Of the 545 *S. flexneri* strains tested, 99.3% contained genes that encode SPATE proteins, such as class II (SepA, Pic) and/or class I (SigA, Sat) (Table 3). A total of 35.8% of the strains had only one or two class I SPATE genes and no class II SPATE. No strains had only class II SPATE, and 54.5% had both two class I SPATE genes and two class II SPATE genes. The most common SPATE among *S. flexneri* strains was *sat* (94.1% of strains), following by *sig*A (78.9%), *pic* (77.6%), *sepA* (63.5%). Regarding differences between serotypes, a total of 86.7% *S. flexneri* x had two class I SPATE genes and two class II SPATE genes, following by 72.2% *S. flexneri 4c*, 69.1% *S. flexneri* 2a, 57.5% *S. flexneri* 2b, 38.8% *S. flexneri* 1a, and 0.0% *S. flexneri* 1b.

#### Enterotoxin genes

The *set*1A gene was present in 406(74.5%) *S. flexneri* isolates (198 serotype 2a, 78 serotype 2b, 74 serotype 1a, 10 serotype 1b, 25 serotype x, 16 serotype 4c), and *set*1B was present in 425 (78.0%) *S. flexneri* isolates (206 serotype 2a, 86 serotype 2b, 72 serotype 1a, 6 serotype 1b, 28 serotype x, 17 serotype 4c) (Table 3). Both *set*1A and *set*1B were detected in 375 (68.8%) of *S. flexneri* isolates. The *sen* was present in 310 (56.9%) *S. flexneri* isolates (153 serotype 2a, 61 serotype 2b, 38 serotype 1a, 9 serotype 1b, 22 serotype x, 14 serotype 4c).

#### Fluctuation in time and place

**Fig. 2:**
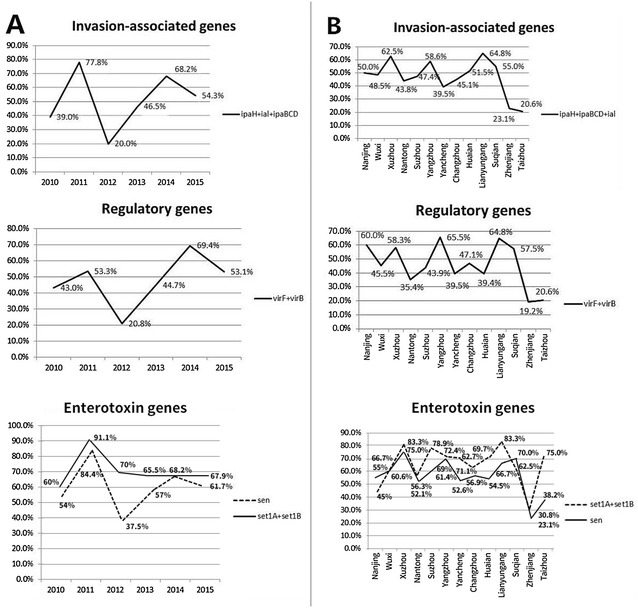
Temporal and regional variation of virulence genes in Jiangsu; **a** temporal variation of virulence genes in Jiangsu during 2010–2015; **b** regional variation of virulence genes in Jiangsu

The existence of these genes had a fluctuation over time and place. In general, there were two epidemic peaks in virulence genes in 2011 and 2014 (Fig. 2). Except for ShET-1, the positivity of virulence genes in *S. flexneri* was the lowest in 2012. There was no regular change in virulence genes between regions, such as the positive rate of invasion-associated genes was the highest in Yangzhou, followed by Lianyungang, but the highest positive rate of ShET-1 existed in Lianyungang, followed by Xuzhou (Fig. 2). When taking into account the different serotypes of year and regional changes, some interesting phenomena were noticed. The positive change in virulence genes of serotype 2a was consistent with the overall change in virulence genes, and serotype 2b had the highest existence of virulence gene in 2013. In addition, the number of virulence genes of serotype 1a isolated in 2012 was obviously smaller than that in other years. It should be noted, however, that the ShET-1 was generally independent of these changes (Additional file 1). Serotype 1a in Zhenjiang was a low virulence gene carrying type. In general, the variation of virulence genes among different serotypes was general not particularly obvious (Additional file 2).

## Discussion

Due to inadequate supply of quality water and low hygienic conditions in less developed countries, *Shigella*—a cause of inflammatory diarrhea and dysentery, poses major challenges to public health sectors. *S. flexneri* was the most common of the four species in many developing countries [19, 20]. However, in developed countries, *S. sonnei* is the commonest *Shigella* species isolated [21, 22]. The reason for this difference is unclear, however, it is apparent that efforts to boost sanitation and local hygiene have greatly decreased the prevalence of shigellosis and even changed the pattern in which *Shigella* species are most distributed. Jiangsu Province is located in the eastern part of China, with a population about 80 million. Epidemiological analysis of *Shigella* will be beneficial to the prevention and control of the infectious diseases in the region. The results of analysis of the distribution characteristics of *S. flexneri* in Jiangsu Province in the present study showed that *S. flexneri* 2a was the most common of the eleven serotype, which is different from the study conducted in Beijing in China reporting that *S. flexneri* 4c was the most prevalent serotype among 19 serotypes [23]. In Jiangsu Province, serotype 4c accounted for only 3.3%. However, our results matched the findings in developing countries [19, 24] and Zhejiang Province of China [25]. Even in Jiangsu Province, there were also differences between the various cities (Additional file 2). For example, most prevalent serotypes in Nanjing are serotypes 2b, serotypes 1b in Zhenjiang and serotypes 1a in Taizhou. What’s more, some rare serotypes were detected at specific times in specific cities, such as serotype 6 was only separated from Nanjing in 2010. High heterogeneity with regard to temporal distribution was noted in *Shigella* species and serotypes, which further suggested the need for serotype-level identification to enhance the effectiveness of control strategies.

Since the information on the variety of *Shigella* virulence genes in China is limited, to fully understand its pathogenicity, further research is required to advance the search for virulence-related genes for *Shigella*. In the present study, the prevalence and distribution of 12 such genes was examined. In the present study, *ipa*H gene was highly conserved in various serotypes. Similar findings have been shown in many other studies [25]. The presence of many copies of this gene i.e. seven in chromosomes and five in plasmids may explain why the gene tested positive in all strains. Considering that this gene can be detected even after the loss of plasmid, it is promising target for diagnostic purposes. In *Shigella,* the ability to enter host cells depends on the availability of type III-secretion-system (T3SS) which are encoded by large virulence plasmids [26, 27]. *ial* gene has been identified in invasion processes and on inv plasmid [28]. Many proteins form part of the T3SS complex which includes a needle-shaped oligomer that connects the inner and outer membrane of the bacteria. The oligomer contains invasive plasmid antigens ipaB, ipaC, and ipaD at its tip end [26–29], which can be identified using upstream region of ipaB, acting as marker. The effects of deleting *ial* and *ipa*BCD on invasiveness of S. *flexneri* are not known. Numerous studies have shown that there is a link between the ability of the *Shigella* spp. strains to cause diarrhea and the presence of invasive genes in the bacteria. Mokhtari et al. [30] showed that, unlike in asymptomatic patients, isolates from stools of patients with diarrhea contained invasive genes, *ial* and/or *ipa*BCD. A study by Phantouamath et al. [31], showed that ial gene was found only in isolates from cases. In our study, 47.0% *S. flexneri*’ isolates were positive for *ial* gene, and 50.5% *S. flexneri*’ isolates were positive for *ipa*BCD gene. Comparison with other similar studies, 78.9% *S. flexneri’* isolates were positive for *ial* gene in Iran [32], and even 100% in Zhejiang of China [25]. For the *ipa*BCD gene, our result is similar to that of a study in Peru (49%) [19], but lower than that of a study in Brazil (100%) [33]. in this sense, the invasive ability of *S. flexneri* in Jiangsu Province is not strong compared with other areas. Moreover, prevalence of virulence genes showed obvious serotype characteristics, such as none *S. flexneri* 1b expressed both *ial* and *ipa*BCD strains. But it should be noted that the pathogenicity of *S. flexneri* is also related to both the number of infected bacteria and the immunity of infected people.

Expression of *Shigella* virulence genes is regulated by heat-stable nucleoid structural protein (H-NS) which downregulates their transcription during unfavorable conditions for invasion. In response to favorable environmental signals, transcription of a series of genes is activated starting from AraC-like protein gene virF, which subsequently turns on transcription of *vir*B regulatory genes. Thereafter, virB protein reverses the H-NS-induced inhibition on transcription which eventually turns on the virulence genes on the plasmid [9, 34].In the present study, both *vir*F and *vir*B were found in 45.0% *S. flexneri* isolates, indicating that there might be other pathways for regulating gene expression. In addition, *vir*F but not *vir*B was found in 19.8% *S. flexneri* isolates, suggesting that *vir*F regulated virulence genes not only through *vir*B pathway. Interestingly, of the 545 *S. flexneri*, 11 strains had only *vir*B, which may be due to loss of the *vir*F gene. On the other hand, because of the importance of *vir*F in regulating virulence genes, potential novel antibiotics targeting *vir*F have gained increasing attention [35, 36]. However, only 64.8% of the positive rate of this gene might limit this antibiotics application.

Two new enterotoxins have recently been described in *S. flexneri*. One is called *Shigella* enterotoxin 1 (ShET-1), which is encoded in the *set1* chromosomal gene. It has been suggested that in its active form, the ShET-1 toxin is composed of a subunit A (encoded by set1A) and five B subunits (encoded by set1B) [37]. Other is plasmid-encoded ShET-2 (encoded by sen). ShET-1 and ShET-2 could alter electrolyte and water transport in the small intestine [28], which is closely related to the symptoms of dehydration in the shigellosis. Prior studies reported that *set*1 genes were only detected in S. *flexneri* serotype 2 (2a and 2b) isolates and less so in other serotypes. In contrast, in the current study, many *S. flexneri* serotypes tested positive for *set*1 genes [7, 12, 38]. In some serotypes, however, the prevalence of *set*1 (*set*1A and/or *set*1B) was significantly lower than in other serotypes, such as *S. flexneri* 1b, *S. flexneri* 3b (Table 3). And interestingly, 14.9% of *S. flexneri* had only one subunit of ShET-1, the question about whether a single subunit would affect the pathogenicity of ShET-1 remains to be answered, but which needs further study for verification. The association remains to be further studied. *sen* gene was found in 11 serotypes, with a majority between 40 and 80%, but the serotype 1b positive rate was only 14%. The low positive rate of ShET-1 and ShET-2 in *S. flexneri* 1b means that this serotype has a low ability to cause dehydration.

Another factor that possess virulence activities is the Serine protease autotransporters of Enterobacteriaceae (SPATEs), which are toxins secreted from gram-negative bacteria. Nevertheless, only a few studies have searched for the presence of their encoding genes in large *Shigella* collections. A similar study in Iran found that the sat gene was present in all *S. flexneri* isolates, and the presence of *sig*A, *pic* and *sep*A genes simultaneously were existed in 35.5% of *S. flexneri* [32]. Comparing the similar study, unsurprising, the most common SPATEs among *Shigella* was *sat* in our study, but the positive rate of the other three genes of SPATEs was significantly higher than that of Iran. Interestingly, *sat* is now recognized as a pathogenic *E*. *coli*, although it was initially studied in uropathogenic *E. coli* strains. In comparison with previous studies on the frequencies of *sat* gene in *E. coli* [39, 40], however, the presence of *sat* gene in *Shigella* was found to be higher. It should be noted that except for *sat* gene, SPATEs of serotype 1b was significantly less than that of the other serotypes.

The virulence gene can be used to identify *Shigella*, which had been confirmed by previous studies. Some studies [41, 42] reported that the positive rate of detecting *Shigella* by a PCR assay targeting the *ipa*H gene was higher than that by the traditional culture method. The disadvantage of this method is that it can only identify one virulence gene at a time, though this disadvantage could probably be overcome by multiple PCR techniques by screening the amplified genes in view of the difficulty of multiple PCR and the restriction of the number of amplified genes. *Ipa*H can be used as a marker gene of *Shigella* to detect the *Shigella*. Four genes (*pic*, *set*1A, *set*1B and *sig*A) are located on the chromosome SHI-1 Island, and the *pic* gene overlaps with *set*1A and *set*1B. When *Shigella flexneri set*1A gene was positive for *Shigella flexneri*, 94.1% *Shigella set*1B was positive, and 92.4% *Shigella* isolates were positive for *pic* and *sig*A. *set*1A positive *Shigella* had a stronger representation of the integrity of this segment of the gene. Because of the high expression of *sat* in *Shigella*, the clinical value of its amplification is not significant. Other virulence genes include *ial*, *ipa*BCD, *vir*F, *vir*B, *sen* and *sep*A, all of which are located on the large virulence plasmid (140 MDa). To reflect these virulence genes of *Shigella*, we chose the lowest existent *ial* gene as a marker and found that the positive rate of *ial* positive *S. flexneri*, *ipa*BCD was 98.8%, the positive rate of *vir*F was 96.1%, the positive rate of *vir*B was 92.6%, and the positive rate of *sen* and *sep*A was 94.5%. To sum up, multiplex PCR combination *ipa*H + *set*1A + *ial* can comprehensively reflect the virulence of *Shigella*.

## Conclusion

In the present study, we provided some baseline information about the distribution of some virulence genes in clinical strains of *S. flexneri* in Jiangsu Province in China. It was found that the prevalence of these virulence genes varied greatly, leading to different severities of the disease. The profile of these virulence genes correlated with serotype, period and region. We found a low pathogenicity serotype (1b) and combination between those genes. These findings may help better control and identify *Shigella* strains.

## Additional files

**Additional file 1.** Emporal variation of virulence genes in different serotypes of *S*. *flexneri*.

**Additional file 2.** Regional variation of virulence genes in different serotypes of *S*. *flexneri*.

## Abbreviations

*S. flexneri*: *Shigella flexneri*
ipaH: invasion plasmid antigen H
ial: invasion associated locus
ipaB: invasion plasmid antigen B
set1: Shigella enterotoxin 1
sen: Shigella enterotoxin 2
sat: secreted autotransporter toxin
sigA: Shigella IgA-like protease homologue
CDC: Center for Disease Prevention and Control
