# Supplementary material for: Low distribution of genes encoding virulence factors in Shigella flexneri serotypes 1b clinical isolates from eastern Chinese populations
Source: Gut Pathog. 2017 Dec 16;9:76. doi: 10.1186/s13099-017-0222-9 (PMC5732504; doi:10.1186/s13099-017-0222-9)
Supplement: Supplementary file 1 — Additional file 1. Emporal variation of virulence genes in different serotypes of S. flexneri. [file 13099_2017_222_MOESM1_ESM.docx]

| **F2a** | ipaH | ial | ipaBCD | VirF | VirB | sigA | sepA | sat | pic | set1A | set1B | sen |
| --- | --- | --- | --- | --- | --- | --- | --- | --- | --- | --- | --- | --- |
| 2010(44) | 100.0% | 38.6% | 40.9% | 63.6% | 38.6% | 95.5% | 70.5% | 88.6% | 88.6% | 61.4% | 93.2% | 52.3% |
| 2011(24) | 100.0% | 83.3% | 87.5% | 87.5% | 70.8% | 95.8% | 87.5% | 100.0% | 100.0% | 100.0% | 95.8% | 87.5% |
| 2012(47) | 100.0% | 34.0% | 36.2% | 68.1% | 38.3% | 76.6% | 63.8% | 93.6% | 93.6% | 89.4% | 93.6% | 57.4% |
| 2013(41) | 100.0% | 53.7% | 58.5% | 70.7% | 56.1% | 90.2% | 61.0% | 82.9% | 85.4% | 87.8% | 80.5% | 61.0% |
| 2014(32) | 100.0% | 93.8% | 96.9% | 93.8% | 93.8% | 100.0% | 93.8% | 100.0% | 100.0% | 90.6% | 93.8% | 90.6% |
| 2015(35) | 100.0% | 82.9% | 82.9% | 82.9% | 82.9% | 100.0% | 82.9% | 100.0% | 91.4% | 97.1% | 100.0% | 80.0% |
|  |  |  |  |  |  |  |  |  |  |  |  |  |
| **F2b** | ipaH | ial | ipaBCD | VirF | VirB | sigA | sepA | sat | pic | set1A | set1B | sen |
| 2010(27) | 100.0% | 33.3% | 44.4% | 77.8% | 44.4% | 96.3% | 70.4% | 100.0% | 96.3% | 66.7% | 92.6% | 51.9% |
| 2011(12) | 100.0% | 91.7% | 91.7% | 91.7% | 33.3% | 100.0% | 100.0% | 91.7% | 100.0% | 100.0% | 91.7% | 91.7% |
| 2012(21) | 100.0% | 19.0% | 19.0% | 66.7% | 19.0% | 76.2% | 52.4% | 90.5% | 81.0% | 76.2% | 76.2% | 47.6% |
| 2013(19) | 100.0% | 84.2% | 84.2% | 73.7% | 84.2% | 94.7% | 94.7% | 78.9% | 78.9% | 84.2% | 78.9% | 68.4% |
| 2014(13) | 100.0% | 53.8% | 69.2% | 69.2% | 53.8% | 61.5% | 53.8% | 100.0% | 61.5% | 53.8% | 69.2% | 53.8% |
| 2015(14) | 100.0% | 50.0% | 50.0% | 64.3% | 42.9% | 64.3% | 57.1% | 100.0% | 57.1% | 64.3% | 71.4% | 42.9% |
|  |  |  |  |  |  |  |  |  |  |  |  |  |
| **F1a** | ipaH | ial | ipaBCD | VirF | VirB | sigA | sepA | sat | pic | set1A | set1B | sen |
| 2010(6) | - | - | - | - | - | - | - | - | - | - | - | - |
| 2011(4) | - | - | - | - | - | - | - | - | - | - | - | - |
| 2012(25) | 100.0% | 0.0% | 0.0% | 8.0% | 0.0% | 88.0% | 8.0% | 100.0% | 92.0% | 92.0% | 92.0% | 8.0% |
| 2013(28) | 100.0% | 39.3% | 39.3% | 67.9% | 39.3% | 75.0% | 64.3% | 96.4% | 71.4% | 78.6% | 75.0% | 60.7% |
| 2014(13) | 100.0% | 61.5% | 69.2% | 61.5% | 69.2% | 92.3% | 61.5% | 100.0% | 92.3% | 92.3% | 76.9% | 61.5% |
| 2015(9) | 100.0% | 66.7% | 66.7% | 66.7% | 66.7% | 100.0% | 66.7% | 100.0% | 88.9% | 100.0% | 100.0% | 66.7% |
|  |  |  |  |  |  |  |  |  |  |  |  |  |
| **F1b** | ipaH | ial | ipaBCD | VirF | VirB | sigA | sepA | sat | pic | set1A | set1B | sen |
| 2010(1) | - | - | - | - | - | - | - | - | - | - | - | - |
| 2011(2) | - | - | - | - | - | - | - | - | - | - | - | - |
| 2012(20) | 100.0% | 0.0% | 0.0% | 0.0% | 0.0% | 5.0% | 0.0% | 100.0% | 0.0% | 30.0% | 0.0% |  |
| 2013(10) | 100.0% | 0.0% | 0.0% | 0.0% | 0.0% | 0.0% | 10.0% | 90.0% | 0.0% | 20.0% | 0.0% | 10.0% |
| 2014(12) | 100.0% | 8.3% | 0.0% | 0.0% | 0.0% | 0.0% | 0.0% | 100.0% | 0.0% | 8.3% | 16.7% | 0.0% |
| 2015(19) | 100.0% | 0.0% | 0.0% | 0.0% | 0.0% | 0.0% | 0.0% | 100.0% | 0.0% | 0.0% | 15.8% | 42.1% |
|  |  |  |  |  |  |  |  |  |  |  |  |  |
| **FX** | ipaH | ial | ipaBCD | VirF | VirB | sigA | sepA | sat | pic | set1A | set1B | sen |
| 2010(12) | 100.0% | 50.0% | 50.0% | 100.0% | 58.3% | 100.0% | 75.0% | 100.0% | 100.0% | 58.3% | 100.0% | 75.0% |
| 2011(0) | - | - | - | - | - | - | - | - | - | - | - | - |
| 2012(1) | - | - | - | - | - | - | - | - | - | - | - | - |
| 2013(4) | - | - | - | - | - | - | - | - | - | - | - | - |
| 2014(11) | 100.0% | 90.9% | 100.0% | 100.0% | 100.0% | 100.0% | 100.0% | 100.0% | 100.0% | 100.0% | 81.8% | 100.0% |
| 2015(3) | - | - | - | - | - | - | - | - | - | - | - | - |
