# Supplementary material for: Low distribution of genes encoding virulence factors in Shigella flexneri serotypes 1b clinical isolates from eastern Chinese populations
Source: Gut Pathog. 2017 Dec 16;9:76. doi: 10.1186/s13099-017-0222-9 (PMC5732504; doi:10.1186/s13099-017-0222-9)
Supplement: Supplementary file 2 — Additional file 2. Regional variation of virulence genes in different serotypes of S. flexneri. [file 13099_2017_222_MOESM2_ESM.docx]

| **F2a** | Nanjing(2) | | Wuxi(21) | | Xuzhou(20) | | Changzhou(26) | | Suzhou(25) | Nantong(16) | | Huaian(10) | | Yancheng(12) | | Yangzhou(12) | | Zhenjiang(6) | | Lianyungang(42) | | Suqian(13) | Taizhou(18) |  |  |
| --- | --- | --- | --- | --- | --- | --- | --- | --- | --- | --- | --- | --- | --- | --- | --- | --- | --- | --- | --- | --- | --- | --- | --- | --- | --- |
| ipaH | - | | 100.0% | | 100.0% | | 100.0% | | 100.0% | 100.0% | | 100.0% | | 100.0% | | 100.0% | | - | | 100.0% | | 100.0% | 100.0% |  |  |
| ial | - | | 66.7% | | 55.0% | | 73.1% | | 56.0% | 43.8% | | 60.0% | | 66.7% | | 58.3% | | - | | 71.4% | | 46.2% | 38.9% |  |  |
| ipaBCD | - | | 71.4% | | 60.0% | | 76.9% | | 56.0% | 43.8% | | 60.0% | | 66.7% | | 66.7% | | - | | 71.4% | | 46.2% | 50.0% |  |  |
| virF | - | | 76.2% | | 60.0% | | 84.6% | | 64.0% | 81.3% | | 60.0% | | 66.7% | | 83.3% | | - | | 81.0% | | 76.9% | 66.7% |  |  |
| virB | - | | 61.9% | | 60.0% | | 76.9% | | 48.0% | 37.5% | | 60.0% | | 66.7% | | 66.7% | | - | | 73.8% | | 46.2% | 38.9% |  |  |
| sigA | - | | 100.0% | | 100.0% | | 96.2% | | 88.0% | 87.5% | | 100.0% | | 100.0% | | 75.0% | | - | | 100.0% | | 30.8% | 100.0% |  |  |
| sepA | - | | 81.0% | | 75.0% | | 73.1% | | 64.0% | 62.5% | | 80.0% | | 58.3% | | 75.0% | | - | | 90.5% | | 61.5% | 66.7% |  |  |
| sat | - | | 100.0% | | 80.0% | | 92.3% | | 80.0% | 100.0% | | 100.0% | | 100.0% | | 83.3% | | - | | 97.6% | | 92.3% | 100.0% |  |  |
| pic | - | | 90.5% | | 90.0% | | 88.5% | | 88.0% | 87.5% | | 100.0% | | 91.7% | | 83.3% | | - | | 100.0% | | 92.3% | 94.4% |  |  |
| set1A | - | | 100.0% | | 80.0% | | 92.3% | | 88.0% | 81.3% | | 70.0% | | 91.7% | | 75.0% | | - | | 92.9% | | 92.3% | 72.2% |  |  |
| set1B | - | | 95.2% | | 90.0% | | 92.3% | | 92.0% | 81.3% | | 80.0% | | 100.0% | | 75.0% | | - | | 100.0% | | 92.3% | 94.4% |  |  |
| sen | - | | 76.2% | | 70.0% | | 76.9% | | 60.0% | 43.8% | | 60.0% | | 75.0% | | 66.7% | | - | | 73.8% | | 61.5% | 66.7% |  |  |
| **F2b** | | Nanjing(5) | | Wuxi(3) | | Xuzhou(16) | | Changzhou(4) | | | Suzhou(10) | | Nantong(15) | | Huaian(19) | | Yancheng(10) | | Yangzhou(11) | | Zhenjiang(4) | | Lianyungang(1) | Suqian(8) | Taizhou(0) |
| ipaH | | - | | - | | 100.0% | | - | | | 100.0% | | 100.0% | | 100.0% | | 100.0% | | 100.0% | | - | | - | - | - |
| ial | | - | | - | | 75.0% | | - | | | 50.0% | | 40.0% | | 52.6% | | 30.0% | | 54.5% | | - | | - | - | - |
| ipaBCD | | - | | - | | 75.0% | | - | | | 40.0% | | 40.0% | | 52.6% | | 50.0% | | 54.5% | | - | | - | - | - |
| virF | | - | | - | | 87.5% | | - | | | 100.0% | | 60.0% | | 52.6% | | 80.0% | | 81.8% | | - | | - | - | - |
| virB | | - | | - | | 75.0% | | - | | | 10.0% | | 26.7% | | 52.6% | | 40.0% | | 54.5% | | - | | - | - | - |
| sigA | | - | | - | | 93.8% | | - | | | 60.0% | | 53.3% | | 100.0% | | 90.0% | | 90.9% | | - | | - | - | - |
| sepA | | - | | - | | 81.3% | | - | | | 90.0% | | 60.0% | | 68.4% | | 80.0% | | 72.7% | | - | | - | - | - |
| sat | | - | | - | | 100.0% | | - | | | 90.0% | | 93.3% | | 73.7% | | 100.0% | | 100.0% | | - | | - | - | - |
| pic | | - | | - | | 100.0% | | - | | | 80.0% | | 53.3% | | 89.5% | | 80.0% | | 90.9% | | - | | - | - | - |
| set1A | | - | | - | | 93.8% | | - | | | 70.0% | | 46.7% | | 94.7% | | 70.0% | | 72.7% | | - | | - | - | - |
| set1B | | - | | - | | 100.0% | | - | | | 80.0% | | 60.0% | | 78.9% | | 90.0% | | 90.9% | | - | | - | - | - |
| sen | | - | | - | | 87.5% | | - | | | 60.0% | | 40.0% | | 57.9% | | 60.0% | | 63.6% | | - | | - | - | - |
|  | |  | |  | |  | |  | | |  | |  | |  | |  | |  | |  | |  |  |  |
| **F1a** | | Nanjing(0) | | Wuxi(4) | | Xuzhou(5) | | Changzhou(3) | | | Suzhou(9) | | Nantong(11) | | Huaian(3) | | Yancheng(5) | | Yangzhou(0) | | Zhenjiang(15) | | Lianyungang(6) | Suqian(8) | Taizhou(39) |
| ipaH | | - | | - | | - | | - | | | 100.0% | | 100.0% | | - | | - | | - | | 100.0% | | - | - | 100.0% |
| ial | | - | | - | | - | | - | | | 55.6% | | 63.6% | | - | | - | | - | | 0.0% | | - | - | 0.0% |
| ipaBCD | | - | | - | | - | | - | | | 55.6% | | 72.7% | | - | | - | | - | | 0.0% | | - | - | 2.6% |
| virF | | - | | - | | - | | - | | | 66.7% | | 100.0% | | - | | - | | - | | 0.0% | | - | - | 15.4% |
| virB | | - | | - | | - | | - | | | 55.6% | | 54.5% | | - | | - | | - | | 0.0% | | - | - | 2.6% |
| sigA | | - | | - | | - | | - | | | 55.6% | | 100.0% | | - | | - | | - | | 6.7% | | - | - | 84.6% |
| sepA | | - | | - | | - | | - | | | 66.7% | | 100.0% | | - | | - | | - | | 0.0% | | - | - | 15.4% |
| sat | | - | | - | | - | | - | | | 100.0% | | 90.9% | | - | | - | | - | | 100.0% | | - | - | 100.0% |
| pic | | - | | - | | - | | - | | | 66.7% | | 81.8% | | - | | - | | - | | 0.0% | | - | - | 84.6% |
| set1A | | - | | - | | - | | - | | | 66.7% | | 100.0% | | - | | - | | - | | 40.0% | | - | - | 89.7% |
| set1B | | - | | - | | - | | - | | | 77.8% | | 81.8% | | - | | - | | - | | 13.3% | | - | - | 84.6% |
| sen | | - | | - | | - | | - | | | 66.7% | | 81.8% | | - | | - | | - | | 0.0% | | - | - | 15.4% |
|  | |  | |  | |  | |  | | |  | |  | |  | |  | |  | |  | |  |  |  |
| **F1b** | | Nanjing(3) | | Wuxi(4) | | Xuzhou(0) | | Changzhou(12) | | | Suzhou(2) | | Nantong(3) | | Huaian(3) | | Yancheng(0) | | Yangzhou(3) | | Zhenjiang(1) | | Lianyungang(4) | Suqian(5) | Taizhou(39) |
| ipaH | | - | | - | | - | | 100.0% | | | - | | - | | - | | - | | - | | - | | - | - | 100.0% |
| ial | | - | | - | | - | | 0.0% | | | - | | - | | - | | - | | - | | - | | - | - | 0.0% |
| ipaBCD | | - | | - | | - | | 0.0% | | | - | | - | | - | | - | | - | | - | | - | - | 2.6% |
| virF | | - | | - | | - | | 0.0% | | | - | | - | | - | | - | | - | | - | | - | - | 15.4% |
| virB | | - | | - | | - | | 0.0% | | | - | | - | | - | | - | | - | | - | | - | - | 2.6% |
| sigA | | - | | - | | - | | 0.0% | | | - | | - | | - | | - | | - | | - | | - | - | 84.6% |
| sepA | | - | | - | | - | | 0.0% | | | - | | - | | - | | - | | - | | - | | - | - | 15.4% |
| sat | | - | | - | | - | | 100.0% | | | - | | - | | - | | - | | - | | - | | - | - | 100.0% |
| pic | | - | | - | | - | | 0.0% | | | - | | - | | - | | - | | - | | - | | - | - | 84.6% |
| set1A | | - | | - | | - | | 8.3% | | | - | | - | | - | | - | | - | | - | | - | - | 89.7% |
| set1B | | - | | - | | - | | 8.3% | | | - | | - | | - | | - | | - | | - | | - | - | 84.6% |
| sen | | - | | - | | - | | 0.0% | | | - | | - | | - | | - | | - | | - | | - | - | 15.4% |
